# Effects of Chemotherapy on Aortic 18-Fluorodeoxyglucose Uptake in Patients With Hodgkin and Non-Hodgkin Lymphoma

**DOI:** 10.1016/j.jacadv.2023.100277

**Published:** 2023-03-31

**Authors:** Charalambos V. Vlachopoulos, Eirini G. Solomou, Dimitrios G. Terentes Printzios, Anastasia G. Pouli, Anastasia Sioni, Stavroula E. Giannouli, Maria K. Angelopoulou, Pavlos Kafouris, Marinos G. Metaxas, Spiros D. Chondropoulos, Ioanna E. Stergiou, Theodoros P. Marinakis, Iosif Koutagiar, Antigoni A. Miliou, Nikolaos Ioakeimidis, Sotirios T. Tsalamandris, Vasiliki Katsi, Constantina I. Aggeli, Michael Voulgarelis, Dimitrios M. Tousoulis, Constantinos Tsioufis, Constantinos D. Anagnostopoulos

**Affiliations:** a 1st Department of Cardiology, Medical School, National and Kapodistrian University of Athens, Hippokration Hospital, Athens, Greece; b Department of Hematology, “Aghios Savvas” Hospital, Athens, Greece; c 2nd Department of Internal Medicine, Medical School, National and Kapodistrian University of Athens, Athens, Greece; d Department of Hematology, General Hospital “LAIKO”, Athens, Greece; e Department of Informatics and Telecommunications, National and Kapodistrian University of Athens, Athens, Greece; f Center of Systems Biology, Biomedical Research Foundation of the Academy of Athens, Athens, Greece; g Department of Pathophysiology, School of Medicine, University of Athens, Athens, Greece; h Department of Clinical Hematology, Georgios Gennimatas Hospital, Athens, Greece; i Biochemistry, Immunology and Molecular Biology Department, National and Kapodistrian University of Athens, Hippokration Hospital, Athens, Greece; j Center for Experimental Surgery, Clinical and Translational Research, Biomedical Research Foundation, Academy of Athens, Athens, Greece

**Keywords:** 1st line treatment response, aortic inflammation, chemotherapy, Hodgkin lymphoma, non-Hodgkin lymphoma, positron emission tomography

## Abstract

**Background:**

Despite advances in the treatment of oncology patients, therapy-related side effects may lead to premature morbidity. Inflammatory activation that has been linked to cardiovascular disease is crucial for the pathogenesis of both Hodgkin (HL) and non-Hodgkin lymphoma (NHL).

**Objectives:**

The purpose of this study was to assess the vascular effects of chemotherapy in patients with HL and NHL by positron emission tomography/computed tomography with 18-fluorodeoxyglucose (18-FDG PET/CT) and to investigate interactions with systemic inflammation as assessed by circulating inflammatory markers.

**Methods:**

Between July 2015 and July 2019, 65 consecutive patients (mean age 56 ± 17.78 years) with confirmed diagnosis of either HL (n = 33) or NHL (n = 32) were prospectively studied. PET/CT imaging was performed at baseline, at an interim phase, and after first-line treatment. Aortic FDG uptake was assessed by measuring global aortic target-to-background ratio (GLA-TBR). Serum biomarkers interleukin (IL)-6 and IL-1b were measured at each phase.

**Results:**

Patients with HL demonstrated significant reduction in aortic TBR after first-line treatment (median GLA-TBR baseline: 1.98, median GLA-TBR third scan: 1.75, median difference = −0.20, 95% CI: −0.07 to −0.33, *P* = 0.006), which remained significant after adjustment for confounders (adj. R^2^ of model = 0.53). In contrast, patients with NHL did not demonstrate a significant aortic inflammation response (*P* = 0.306). Furthermore, patients with HL demonstrated a significant reduction in IL-6 (*P* = 0.048) and IL-1b (*P* = 0.045), whereas patients with NHL did not demonstrate significant reduction in IL-6 (*P* = 0.085) and IL-1b levels (*P* = 0.476).

**Conclusions:**

Aortic inflammation, as assessed by 18-FDG PET/CT, is reduced in HL patients after first-line treatment but not in NHL patients. These findings imply that different pathophysiological pathways and different therapies might affect the arterial bed in different ways for patients with lymphoma.

Despite significant advances in the treatment of oncology patients that have resulted in significant improvement of survival, therapy-related side effects may lead to premature morbidity and death among cancer survivors.^1^^,^^2^ Concerns relate mainly to accelerated development of coronary artery disease or even direct effects of treatment on the heart muscle. However, despite intensive research on the heart itself, there is little evidence of the effect of chemotherapy on larger arteries and peripheral vasculature, the integrity of which plays an important role in cardiovascular risk and cardiac function.^3^^,^^4^

Inflammatory activation drives many malignancy types and is particularly crucial for the pathogenesis of both Hodgkin (HL) and non-Hodgkin lymphoma (NHL).^5^ Several markers of inflammation that have been associated with disease activity and prognosis in lymphoma patients are also associated with cardiovascular diseases (CVDs).^6^, ^7^, ^8^, ^9^ Positron emission tomography/computed tomography with 18-fluorodeoxyglucose (FDG PET/CT) is a versatile tool with an established role in staging malignancies including lymphomas. In addition, it is a reliable and reproducible measure of vascular inflammation by measuring arterial wall FDG uptake, increased values of which have been associated with future cardiovascular events.^10^ Moreover, in the COVID-19 era, we have recently shown that aortic 18 FDG PET/CT increases transiently in patients with severe or critical COVID-19, offering the potential to serve as a predictor of outcome in these patients.^11^

Because lymphoma chemotherapy has been deemed potentially cardiotoxic, one can infer that it may have similar unfavorable effects on arterial inflammation.^12^ However, previous studies from our and other laboratories have shown a reduction of aortic FDG uptake after treatment of pathologies that cause chronic inflammation.^13^^,^^14^ Furthermore, we have recently shown that aortic wall FDG uptake is related with disease severity in patients with both HL and NHL.^15^ Hence, we hypothesized that favorable lymphoma response to treatment may be associated with beneficial effects on vascular inflammation. Therefore, in the present study, we assessed the vascular effects of chemotherapy in patients with HL and NHL by 18 FDG PET/CT and investigated possible interactions with systemic inflammation as assessed by circulating inflammatory markers.

## Materials and methods

### Study population

Between July 2015 and July 2019, 65 consecutive patients from 3 hematology centers were prospectively included in the study. These patients had histologically confirmed new diagnosis of lymphoma and gave written informed consent to undergo 3 imaging sessions with PET/CT scan with 2 scans per session (1 clinically indicated at 60 minutes, and a second one at 120 minutes for assessment of aortic uptake). PET/CT imaging was performed at baseline, at an interim phase, and after first-line treatment. Specifically, for patients with HL, the interim scan was obtained at 1 to 3 days prior to initiating the third chemotherapy cycle, while for patients with NHL, 2 weeks after the fourth chemotherapy cycle. All patients were reassessed 6 to 8 weeks after chemotherapy completion. Chemotherapy completion was defined as the completion of first-line treatment.

Patients with HL received the ABVD scheme (doxorubicin, bleomycin, vinblastine, dacarbazine), while patients with NHL received the RCHOP scheme (rituximab, cyclophosphamide, doxorubicin hydrochloride, vincristine, prednisolone).

Sociodemographic data, history of cerebrovascular and cardiovascular events, cardiovascular risk factors, and current medical therapy of participants, as well as routine laboratory tests, were collected for all subjects. Family history of CVDs included history of acute myocardial infarction, nonembolic stroke, and dyslipidemia; the latter was defined if the patient had already been categorized as dyslipidemic or was already on treatment for dyslipidemia. The Framingham 10-year general CVD risk score (using their body mass index) was used to assess each patient’s CVD risk.^16^^,^^17^ Exclusion criteria were recent (<6 months) cardiovascular event, aortitis, active infection or systemic autoimmune disease, venous thromboembolic disease, renal failure, and treatment with anti-inflammatory agents. Patients with inadequate images for analysis owing to excessive spillover within the arterial wall from surrounding nodal or extranodal FDG uptake were also excluded.

Patients were anonymized and had a unique serial 5-digit number. Their PET scans and blood samples were labelled with their personal 5-digit number. A further characterization with B, I, and P, which stand for baseline, interim, and post first-line treatment, was applied accordingly. Both the patient’s ID and the time that the scan was taken or the blood samples were collected were unknown to the scan readers and to our lab technicians before their analyses. Subsequently, and when the protocol ended, scans were assessed within a month by the 2 blinded readers in a random order irrespective of the patient’s ID or the time of assessment. Blood samples were stored in −81 °C and were all assessed in a single batch.

The study protocol was approved by the institutional research ethics committee of each participating center, and the study was conducted according to institutional guidelines and the Declaration of Helsinki.

### FDG PET/CT imaging

All participants underwent FDG PET/CT imaging within 7 days before the initiation of chemotherapy, after fasting for at least 6 hours prior to the study. None of the patients had blood glucose levels >180 mg dL^−1^ before injection. FDG was injected intravenously (5 MBq/kg), and scanning was performed at 60 and 120 minutes after injection for disease staging evaluation and aortic tracer uptake assessment, respectively. Patients were scanned from the base of the skull to the upper third of the thighs on a hybrid PET/CT scanner (Biograph 6; Siemens). For the second scan, acquisition was restricted to the thoracic and abdominal region. A low-dose CT scan in supine position was obtained, with patients’ arms placed above their heads. No CT intravenous contrast was administered. CT images were acquired with 30 mA, 130 KV, an axial slice thickness of 5 mm, and table feed rotation of 27 mm per tube rotation. PET scanning followed immediately over the same predefined body region, and the images were reconstructed using a standard iterative ordered-subset expectation maximization algorithm. The image reconstruction matrix employed was 168 × 168. The reconstruction scheme of choice in this work consisted of 4 iterations and 8 subsets. PET images were acquired for 6 minutes per bed position.

Treatment response was assessed according to the revised Lugano criteria for malignant lymphoma, using the Deauville 5-point scale for FDG uptake by PET/CT^18^; score 1: no uptake above background, score 2: uptake ≤ mediastinum, score 3: uptake > mediastinum but ≤ liver; score 4: uptake moderately > liver, score 5: uptake markedly higher than liver and/or new lesions. Briefly, complete metabolic response was defined as score 1, 2, or 3 with or without a residual mass, partial metabolic response as score 4 or 5 with reduced uptake compared with baseline and residual masses of any size, stable disease as score 4 or 5 with no significant change in FDG uptake from baseline, and progressive disease as score 4 or 5 with an increase in intensity of uptake from baseline and/or new FDG-avid lesions. For the purposes of this study, we considered patients with Deauville scores ≤3 as patients with complete metabolic response at the end of first-line treatment, while patients with Deauville scores >3 were considered to have either incomplete or no metabolic response at the end of first-line treatment. Deauville scores of all patients' scan after first-line treatment are shown in Table 1.

**Table 1:** Patient Characteristics

	Total Patients (N = 65)	Non-Hodgkin (n = 32)	Hodgkin (n = 33)	*P* Value
Age (y)	56.03 ± 17.78	62.06 (±15.17)	50.18 (±18.39)	**0.006**
Men	56.9% (n = 37)	56.3% (n = 18)	57.6% (n = 19)	0.915^a^
Weight (kg)	78 ± 16.64	80.06 ± 16.31	76 ± 16.97	0.329
Height (cm)	169 ± 9.07	168.78 ± 9.26	169 ± 9.01	0.819
BMI, kg/m^2^	27.24 ± 5.52	28.19 ± 5.92	26.32 ± 5	0.173
Risk factors				
Diabetes	4 (6.2%)	9.4% (n = 3)	3% (n = 1)	0.291^a^
Hypertension	19 (29.2%)	43.8% (n = 14)	15.2% (n = 5)	**0.012**^a^
Dyslipidemia	24.2% (n = 16)	40.6% (n = 13)	9.1%(n = 3)	**0.003**^a^
Smokers	21.5% (n = 14)	15.6% (n = 5)	27.3% (n = 9)	0.952^a^
Ex-smokers	10.8% (n = 7)	12.5% (n = 4)	9.1% (n = 3)	
Framingham risk score^b^	16 (4.85-30.85)	18.8 (7.8-37.72)	16.14 (2.3-25.8)	**0.039**
Family history of CAD	15.4% (n = 10)	3.1% (n = 1)	27.3% (n = 9)	**0.007**^a^
Deauville score in scan after first-line treatment				
1	49.2% (n = 32)	50% (n = 16)	48.5% (n = 16)	-
2	30.8% (n = 20)	25% (n = 8)	36.4% (n = 12)	-
3	1.5% (n = 1)	3.1% (n = 1)	3% (n = 1)	-
4	3.1% (n = 2)	3.1% (n = 1)	3% (n = 1)	-
5	15.4% (n = 10)	21.9% (n = 7)	9.1% (n = 5)	-
Medications				
Aspirin	9.2% (n = 6)	6.3% (n = 2)	12.1% (n = 4)	0.417
ADP inhibitors	4.6% (n = 3)	6.3% (n = 2)	3% (n = 1)	0.539
ACEI	9.2% (n = 6)	15.6% (n = 5)	3% (n = 1)	0.082
ARBs	9.2% (n = 6)	21.9% (n = 7)	9.1% (n = 3)	0.067
Beta-blockers	16.9% (n = 11)	18.8% (n = 6)	15.2% (n = 5)	0.701
Calcium antagonists	9.2% (n = 6)	12.5% (n = 4)	6.1% (n = 2)	0.374
Statins	18.5% (n = 12)	28.1% (n = 9)	9.1% (n = 3)	0.057

Values are mean ± SD, %, or median (25th-75th percentile). Statistically significant differences are in **bold**. *P* < 0.05 for all between-group comparisons.

ACEI = angiotensin-converting enzyme inhibitors; ADP = adenosine diphosphate receptor; ARB = angiotensin II receptor blocker; BMI = body mass index; CAD = cardiovascular disease.

a Fisher’s exact value.

b The Framingham 10-year general cardiovascular disease (CVD) risk score (using their BMI) was used to assess each patient’s 10-year CVD risk.

### Aortic FDG uptake assessment

FDG PET/CT images were assessed in consensus by 2 investigators with experience in cardiovascular PET/CT image analysis (E.G.S. and P.K.) without the knowledge of patients’ data. The aortic FDG uptake quantification has been previously described.^19^^,^^20^ In brief, regions of interest (ROIs) around the aortic wall were manually drawn along the entire aorta in consecutive axial slices at intervals of 5 mm. Metabolic activity within each arterial ROI was measured by the maximum standardized uptake value (SUV). In the next step, 6 consecutive circular ROIs of 3 mm diameter were drawn within the superior vena cava, and an average venous SUVmean value was calculated. The arterial target-to-background ratio (TBR) was then derived by dividing the mean aortic maximum SUV by the average value of venous SUVmean. Finally, global aortic TBR (GLA-TBR) was calculated as the sum of TBRs of ascending and descending aorta, aortic arch, and suprarenal and infrarenal abdominal aorta divided by 5.

### Evaluation of systemic inflammation

Before FDG PET/CT imaging, blood was obtained, and serum was separated by centrifugation at 4000 rpm for 10 minutes at 4 °C and stored at −81 °C. Serum biomarkers interleukin (IL)-6 and IL-1b were measured at baseline and after treatment in all patients.

### Statistical analysis

Quantitative data are presented as mean ± SD or median (IQR), while qualitative variables are presented as absolute and relative frequencies. The assumptions for linearity and homoscedasticity were tested based on the standardized residuals plots, whereas the assumption of normality for the dependent variable was tested by using the Kolmogorov-Smirnov criterion. Logarithmic transformation was performed for distributions that were significantly skewed before analysis (aortic TRB, IL-6, IL-1b). Aortic TBR had a good fit to normal distribution after log transformation, is expressed as a median (25th-75th percentile), and was analyzed with parametric tests. For skewed variables even after log transformation (IL-6, IL-1b), nonparametric tests were applied for comparison between different time points.

Chi-square test was used for comparison between groups of categorical variables. Student *t*-tests for symmetrical continuous variables and Mann-Whitney U tests for skewed continuous variables were used for comparisons between 2 groups. For comparison of values at the same group before and after treatment, paired *t*-test was used in symmetrical variables, and Wilcoxon matched-pairs sign-ranks test in skewed variables.

For aortic TBR, between different time points, comparisons were done by using a repeated-measures analysis of variance. Multivariable analysis was performed by linear multiple regression analysis. Log-transformed aortic TBR was used as the dependent variable, and baseline aortic TBR, 10-year cardiovascular risk assessed by Framingham risk score, dyslipidemia, and diabetes as independent variables and covariates. The covariates were chosen based on previous literature, suggesting that increased aortic TBR is associated with increased cardiovascular risk (10). A 2-tailed *P* value <0.05 was considered significant. All statistical analyses were performed with the SPSS 24.0 (SPSS Inc).

Intraobserver and interobserver variability assessments for PET/CT image analysis are further analyzed, and the results are provided in Supplemental Material (see also Supplemental Figure 1).

## Results

### Study population and baseline assessment

The demographic, clinical, and laboratory characteristics of all participants are summarized in Table 1. In total, 65 consecutive patients (37 males, mean age 56.03 ± 17.78 years) with HL (n = 33) or NHL (n = 32) were enrolled in the study. Patients with NHL were older and more often with a diagnosis of dyslipidemia and hypertension. A positive family history of CAD was more often seen in patients with HL. The Framingham risk score calculated using body mass index was higher in patients with NHL than that in HL patients (*P* = 0.039).

Aortic logTBR at baseline was similar between patients with NHL and those with HL (median TBR_NHL_ = 2.0 [IQR: 1.79-2.31] vs median TBR_HL_ = 1.94 [IQR: 1.67-2.17], *P* = 0.167).

### End of treatment metabolic response assessment

In the HL group, 30 patients (90.9%) demonstrated complete metabolic response, while 3 (9.1%) patients demonstrated incomplete or no metabolic response. In the NHL group, the corresponding numbers were 25 (78.1%) and 7 (21.9%), respectively.

### Effect of first-line treatment on proinflammatory cytokines IL-6 and IL-1b

IL-6 levels were higher in patients with HL than in patients with NHL at baseline (median IL-6 baseline HL = 0.42, [IQR: 0.33-1.73] vs median IL-6 baseline NHL = 0.21, [IQR: 0.13-1.07]; *P* = 0.028). IL-1b was higher in HL patients than that in NHL patients at baseline (median IL-1b baseline HL = 0.65 [IQR: 0.15-1.74] vs median IL-1b baseline NHL = 0.09 [IQR: 0.07-0.49]; *P* = 0.002).

In the whole population, IL-6 demonstrated a significant reduction (median IL-6 before treatment = 0.35 [IQR: 0.15-1.32] vs median IL-6 after first-line treatment = 0.23 [IQR: 0.13-0.85] with median difference = −0.23, 95% CI: −0.06 to −0.75, *P* = 0.005). IL-1b demonstrated a similar response to treatment (median IL-1b before treatment = 0.16 [IQR: 0.08-0.93], median IL-1b after first-line treatment = 0.17 [IQR: 0.07-0.40], with median difference = −0.19, 95% CI −0.38 to −0.002, *P* = 0.048).

**Figure 1:**
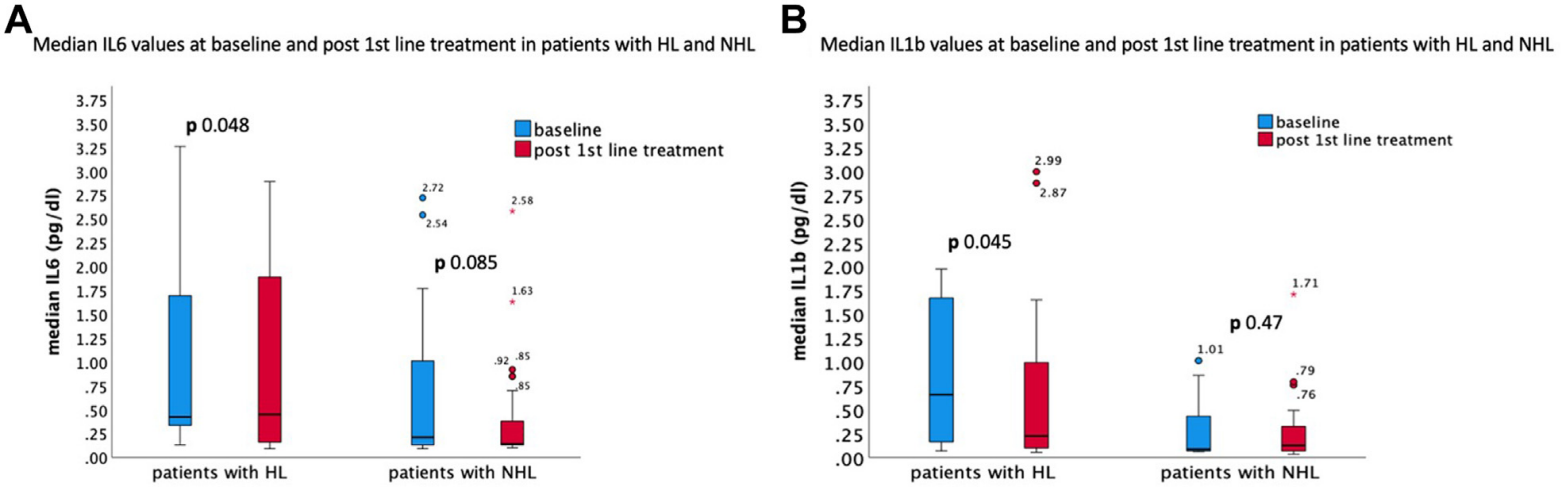
**Median IL-6 and IL-1b Values at Baseline and After First-Line Treatment in Patients With HL and NHL** **(A)** IL-6 at baseline and after first-line treatment in patients with HL and NHL. **(B)** IL-1b at baseline and after first-line treatment in patients with HL and NHL. ∗Values are demonstrated as median (25th-75th percentile). HL = Hodgkin lymphoma; NHL = non-Hodgkin lymphoma.

Patients with HL demonstrated a significant reduction in IL-6 (median difference = −0.28, 95% CI −1.64 to −0.01, *P* = 0.048) and IL-1b (median difference = −0.32, 95% CI −1.06 to −0.01, *P* = 0.045) after first-line treatment in contrast to patients with NHL that did not demonstrate significant reduction in IL-6 (median difference = −0.19, 95% CI −0.72 to 0.02, *P* = 0.085) and IL-1b levels (median difference = −0.02, 95% CI −0.32 to 0.07, *P* = 0.476) (Figure 1).

### Effect of treatment on aortic TBR

#### Overall population

**Figure 2:**
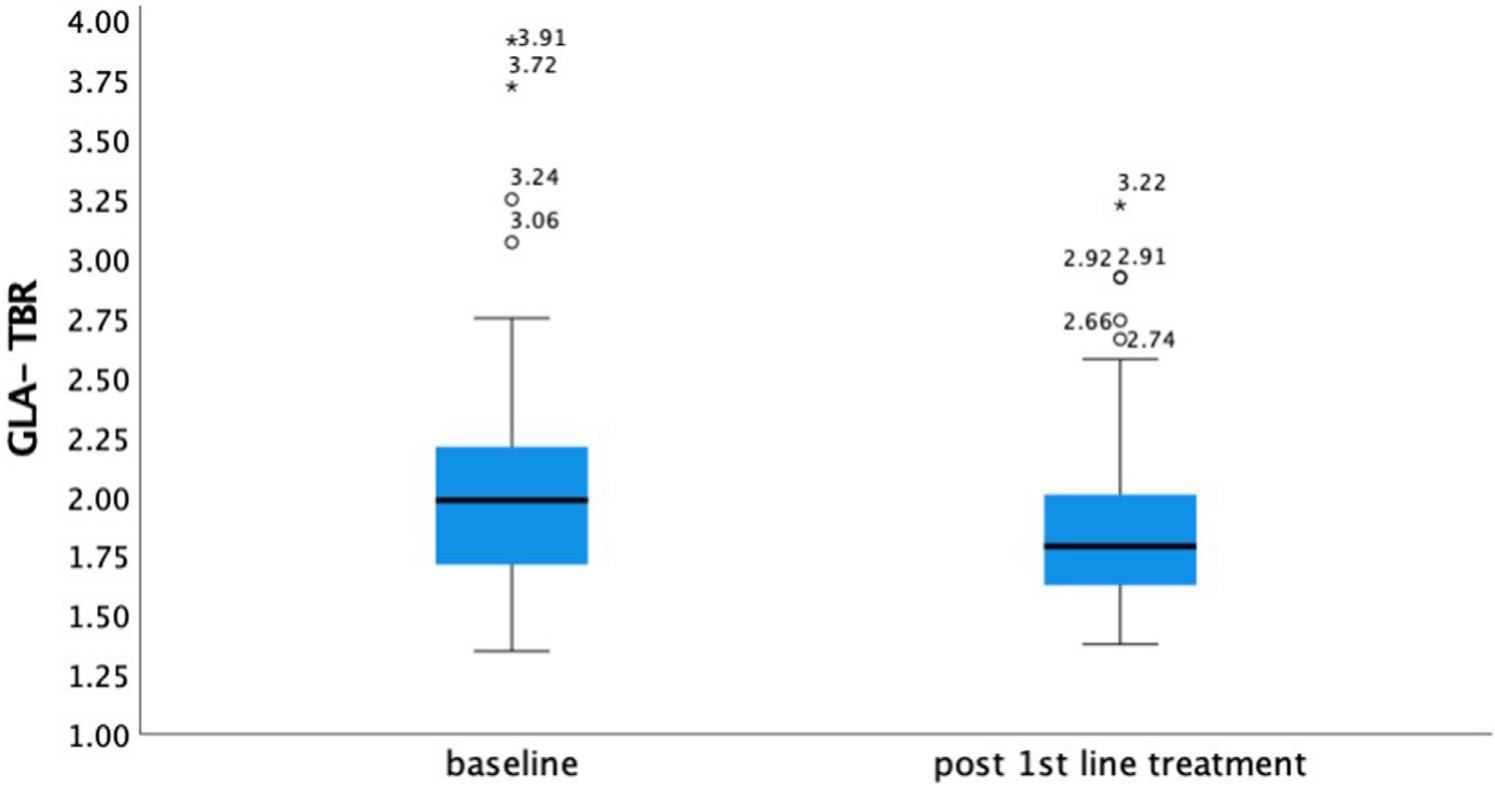
**Box Plot of Median GLA-TBR at Baseline During and After First-Line Treatment for Patients With Lymphoma** Significant reduction of GLA-TBR in patients with lymphoma before and after completion of first-line treatment. The **asterisks** is suggesting values of patients that are extreme outliers, while the **circles** are values of patients that are outliers. GLA-TBR = global aortic target-to-background ratio.

There was a statistically significant reduction in GLA-TBR (median GLA-TBR_baseline_: 1.98 [25th-75th percentile: 1.70-2.21], median GLA-TBR_3rd scan_: 1.78 [25th-75th percentile 1.62-2.00]) when comparing baseline to the end-of-treatment values (median difference = −0.16, 95% CI: −0.03 to −0.30, *P* = 0.013). The GLA-TBR change during treatment in patients with lymphoma is demonstrated in Figure 2. Similarly, there was a significant reduction of GLA-TBR (median difference = −0.16, 95% CI: −0.04 to −0.28, *P* = 0.008), following exclusion of patients without an incomplete metabolic response. GLA-TBR change during treatment in patients with HL is depicted in Supplemental Figure 2.

#### Patients with HL

Ιn the group of patients with HL, there was a statistically significant reduction in GLA-TBR (median GLA-TBR_baseline_: 1.98 [IQR: 1.67-2.17], median GLA-TBR_3rd scan_: 1.75 [IQR: 1.53-1.92]) when comparing baseline to the end-of-treatment scans (median difference = −0.20, 95% CI: −0.07 to −0.33, *P* = 0.006). In linear multivariable regression analysis, GLA-TBR reduction remained statistically significant (*P* = 0.001, t = −3.35), after adjustment for GLA-TBR at baseline (*P* < 0.001, t = 7.74), 10-year cardiovascular risk (Framingham risk score), dyslipidemia, hypertension, and diabetes (R^2^ of model = 0.57, adj. R^2^ of model = 0.53) (Table 2).

**Table 2:** Multivariate Analysis

Positive Variables Selected	*b* (95% CI)	SE b	*t*	*P* Value
(Dependent variable = GLA-TBR), adjusted R^2^ = 0.53				
Framingham CVD risk score (using BMI)	−0.000 (−0.001 to 0.001)	0.001	−0.331	0.742
Baseline GLA-TBR	−0.043 (−0.069 to −0.017)	0.081	7.742	**<0.001**
TBR change from baseline to after the first-line treatment	0.107 (0.057 to 0.157)	0.013	−3.354	**0.001**
Diabetes	0.010 (−0.10 to 0.12)	0.055	0.179	0.858
Hypertension	−0.015 (−0.058 to 0.028)	0.022	−0.699	0.487
Dyslipidemia	0.022 (−0.035 to 0.079)	0.028	0.779	0.439

Statistically significant differences are in **bold**. Linear multivariable regression analysis, demonstrating statistically significant GLA-TBR reduction (*P* = 0.001, t = −3.35), after adjustment for GLA-TBR at baseline (*P* < 0.001, t = 7.74), 10-year cardiovascular risk (Framingham risk score), dyslipidemia, hypertension, and diabetes.

BMI = body mass index; CI = confidence interval; CVD =cardiovascular disease; GLA-TBR = global aortic target-to-background ratio.

#### Patients with NHL

There was no significant reduction in GLA-TBR after treatment compared to baseline levels (median GLA-TBR_baseline_ 2.13 [IQR: 1.79-2.31], median GLA-TBR_3rd scan_ 2.01 [IQR: 1.66-2.29]) in patients with NHL (*P* = 0.306). When patients without complete metabolic response were excluded, GLA-TBR remained unchanged after treatment (*P* = 0.142). GLA-TBR change in patients with NHL is demonstrated in Supplemental Figure 2. A box plot demonstrating aortic TBR change from baseline to post-first-line treatment level in patients with HL and NHL is demonstrated in Central Illustration.

**Figure 3:**
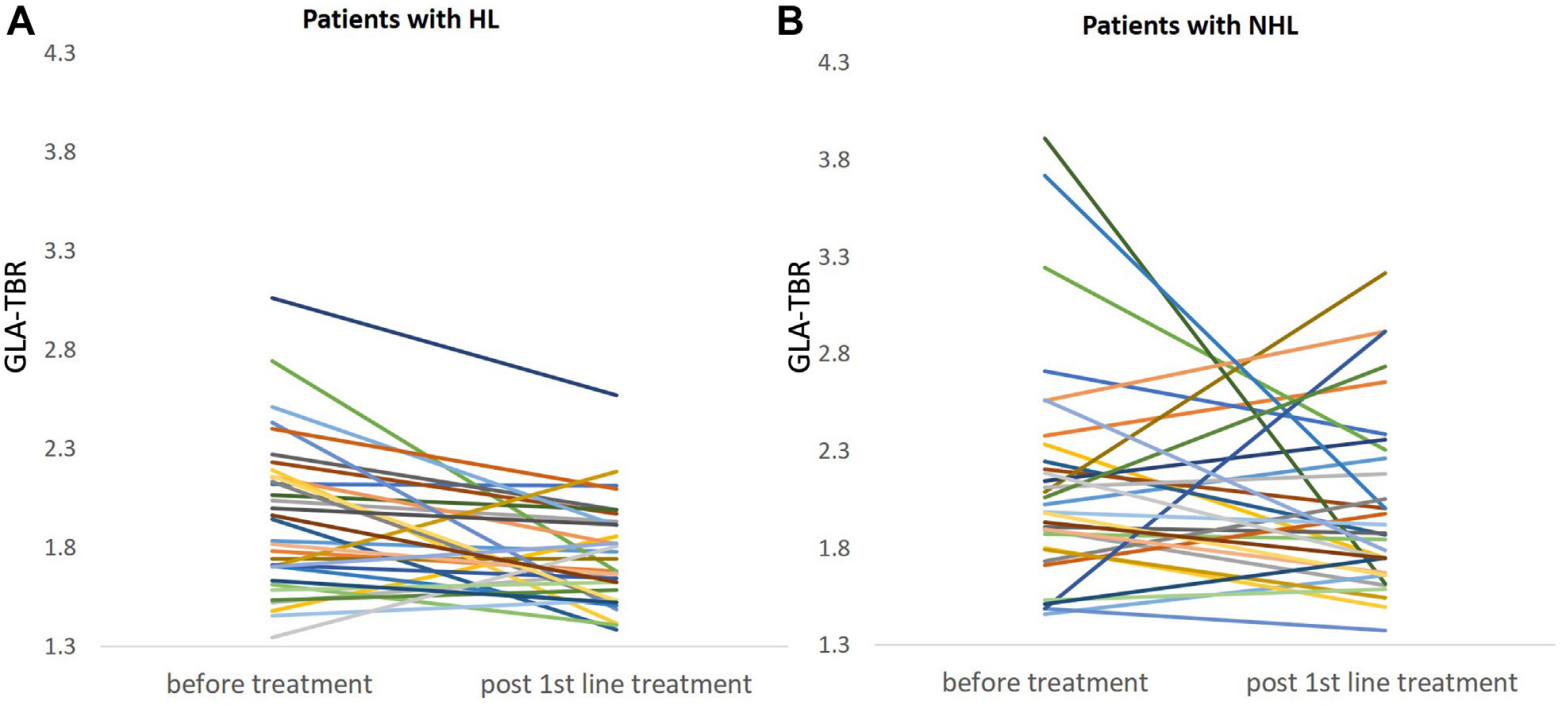
**Line Graphs of Individual GLA-TBR Values at Baseline and After First-Line Treatment in Patients With HL and NHL** **(A)** Individual GLA-TBR values at baseline and after first-line treatment in patients with HL. **(B)** Individual GLA-TBR values at baseline and after first-line treatment in patients with NHL. GLA-TBR = global aortic target-to-background ratio; HL = Hodgkin lymphoma; NHL = non-Hodgkin lymphoma.

**Central Illustration:**
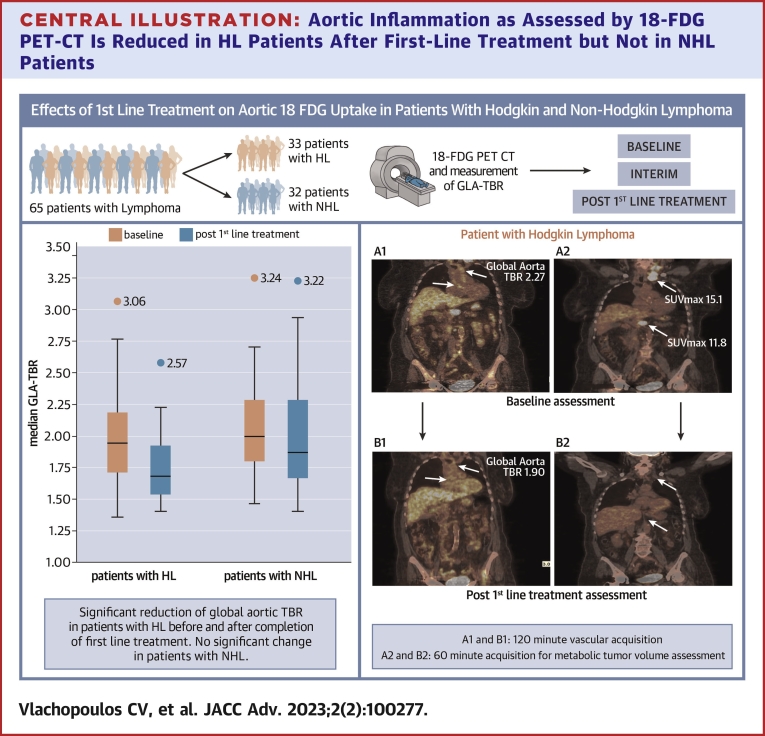
**Aortic Inflammation as Assessed by 18-FDG PET-CT Is Reduced in HL Patients After First-Line Treatment but Not in NHL Patients** These findings imply that different pathophysiological pathways and different therapies might affect the arterial bed in different ways for patients with lymphoma. GLA-TBR = global aortic target-to-background ratio; HL = Hodgkin lymphoma; NHL = non-Hodgkin lymphoma; SUVmax = standard uptake value max.

A further analysis excluding all outliers for all patients and for separate HL and NHL groups is provided in the Supplement.

### Comparison between groups

There was no significant interaction in GLA-TBR for patients with HL and ΝHL with regard to response to first-line treatment (F = 0.79, *P* = 0.37). A paired GLA-TBR line graph of both patients with HL and NHL is demonstrated in Figure 3. An example of a patient with HL with significant aortic inflammation reduction after chemotherapy is demonstrated in Central Illustration.

## Discussion

To the best of our knowledge, this is the first prospective study to evaluate aortic inflammation as assessed by aortic 18-FDG PET/CT in patients with HL and NHL before, during, and after first-line treatment, using a dedicated PET/CT scan protocol. More specifically, while first-line treatment results in significant aortic FDG uptake reduction in patients with HL, there was no such change in patients with NHL; notably, however, there was no significant interaction between the type of lymphoma and aortic inflammation response. Whether these findings are associated with the type of treatment or with individual patient response to treatment needs to be investigated.

### Inflammation in HL and NHL

In our previous study,^15^ we have shown that aortic TBR is related to disease burden in patients with lymphoma. Our current findings extend these results, showing that the initially increased aortic inflammation resolves in patients with HL. There is only 1 relevant retrospective study investigating aortic wall inflammation in patients with HL. Specifically, Lawal et al^21^ assessed 18 FDG PET/CT scans of patients with HL before and after chemotherapy completion (first-line chemotherapy; average time interval until the second scan was 65 weeks). In this study, patients that were assessed earlier than 24 weeks from chemotherapy completion had significantly lower aortic TBR values in all aortic segments than their initial scan. While methodological limitations (retrospective study, incomplete data regarding relapse of the disease, only one 60-minute scan, neither index vessel nor GLA-TBR assessment) hamper solid conclusions, a transient suppression of inflammation in some patients may account for the course of aortic TBR in this group of patients.

The tumor microenvironment of HL, consisting of mononuclear phagocytic cells, is essential for tumor survival. This complex interplay between Reed-Sternberg and reactive cells depends on multiple cytokines and results in inflammatory response. Several markers of inflammation have been associated with disease activity and prognosis in HL, such as IL-6, erythrocyte sedimentation rate, C-reactive protein, and ferritin, and their normalization correlates with response to chemotherapy.^22^ The decline in serum IL-1b and IL-6 after treatment in HL patients in our study is in line with these findings. Anthracyclines promote apoptosis by DNA intercalation and oxidative stress, while alkylating agents promote cell death by inhibition of DNA synthesis. Emerging evidence suggests that in HL, anthracyclines and alkylating agents have potential effects on HL microenvironment, targeting both Reed-Sternberg and the reactive milieu.^6^, ^7^, ^8^

Existing literature supports that both patient groups have increased risk of CVD incidence.^23^^,^^24^ This could be attributed to the disease itself, to possible additional chest radiotherapy, and to the late onset of treatment-related cardiovascular toxicity. However, patients with NHL tend to be of older age and suffer from more comorbidities. Accordingly, these may have accounted for the differences in response in the 2 groups.

### Study Limitations

The present study has some limitations. A modest number of patients with lymphoma were included; however, this was based on a priori power sample size calculation, and our statistical approach was robust.

It is important to note that although both HL and NHL are treated with anthracycline and alkylating agent-based regimes, the NHL group received simultaneous prednisone therapy, which is known to reduce vascular inflammation.

It is unknown how each agent interacts with the vascular bed and whether different dosages could have a different impact on the vascular inflammation response. Regarding specifically the NHL sub-type, in our case, the majority of patients were individuals with diffuse large B cell lymphoma who received RCHOP; hence, the confounding role of this factor is likely to be minimal.

The specific protocol used to assess aortic inflammation requires that the patient receives 2 scans, one at 60 minutes and the other at 120 minutes, which means longer examination time and more radiation exposure due to the second scan. Artificial intelligence algorithms that can extrapolate 60-minute scan results are being evaluated in our lab with promising results; this can facilitate application in clinical practice.

It should also be noted that while our results may point toward specific pathophysiological differences between HL and NHL, they cannot prove causality. Finally, no recommendations can be made for the management of individuals with residual aortic inflammation after first-line treatment. This issue should be targeted in larger prospective multicenter studies.

## Conclusions

We demonstrate for the first time the aortic inflammation response to first-line treatment, as assessed by 18-FDG PET/CT, in patients with HL and NHL. While first-line treatment results in significant aortic FDG uptake reduction in patients with HL, there was no such change in patients with NHL; notably, however, there was no significant interaction effect in the response to treatment between the 2 groups. While these findings imply that lymphoma itself exerts vascular effects, the associations with the type of treatment or with individual patient response to treatment need to be further elucidated. There is a strong potential role of molecular imaging in cardio-oncology; however, further refinements to the technique are needed in order to provide valuable information on disease-related vascular effects and prognosis. Larger prospective studies could provide information regarding the prognostic value of both baseline and residual (after first-line treatment) aortic inflammation in predicting disease relapse and future CVD.

PERSPECTIVES **COMPETENCY IN MEDICAL KNOWLEDGE AND PATIENT CARE:** Our findings may have important clinical implications. Aortic TBR is a prognostic marker of cardiovascular events,^25^, ^26^, ^27^ and its predictive potential may extend to lymphoma patients. Specifically for HL patients, aortic inflammation seems to be disease-related and may, therefore, serve as a marker of disease activity, whereas in NHL, it may not be entirely related to lymphoma activity. However, for both lymphoma types, aortic inflammation could serve as a prognostic marker of long-term CVDs.

**TRANSLATIONAL OUTLOOK:** Overall, our findings suggest that the already routinely available 18 FDG PET/CT scan for lymphoma staging and treatment response assessment could potentially, if applied with a modified protocol, offer an additional surrogate marker, namely GLA-TBR. The latter could be of use for gauging disease severity, progression, and response to treatment, as well as for gauging disease vascular effects. Furthermore, larger prospective studies could provide information regarding the prognostic value of both baseline and residual (after first-line treatment) aortic inflammation in predicting disease relapse and future CVDs.

## Funding support and author disclosures

The study was funded in part by the Hellenic Society of Hypertension. The authors have reported that they have no relationships relevant to the contents of this paper to disclose.

## References

[1] Florido R., Daya N., Ndumele C. (2022). Cardiovascular disease risk among cancer survivors. J Am Coll Cardiol.

[2] Arnold M., Rutherford M.J., Bardot A. (2019). Progress in cancer survival, mortality, and incidence in seven high-income countries 1995–2014 (ICBP SURVMARK-2): a population-based study. Lancet Oncol.

[3] Chirinos J.A., Segers P., Hughes T., Townsend R. (2019). Large-artery stiffness in health and disease: JACC state-of-the-art review. J Am Coll Cardiol.

[4] Vlachopoulos C., Aznaouridis K., Stefanadis C. (2010). Prediction of cardiovascular events and all-cause mortality with arterial stiffness: a systematic review and meta-analysis. J Am Coll Cardiol.

[5] Carbone A., Tripodo C., Carlo-Stella C., Santoro A., Gloghini A. (2014). The role of inflammation in lymphoma. Adv Exp Med Biol.

[6] Lake R.A., Robinson B.W. (2005). Review immunotherapy and chemotherapy--a practical partnership. Nat Rev Cancer.

[7] Suzuki E., Kapoor V., Jassar A.S. (2005). Gemcitabine selectively eliminates splenic Gr-1+/CD11b+ myeloid suppressor cells in tumor-bearing animals and enhances antitumor immune activity. Clin Cancer Res.

[8] Tesniere A., Apetoh L., Ghiringhelli F. (2008). Immunogenic cancer cell death: a key-lock paradigm. Curr Opin Immunol.

[9] Van Eeden S., Leipsic J., Paul Man S.F., Sin D.D. (2012). The relationship between lung inflammation and cardiovascular disease. Am J Respir Care Med.

[10] Paulmier B., Duet M., Khayat R. (2008). Arterial wall uptake of fluorodeoxyglucose on PET imaging in stable cancer disease patients indicates higher risk for cardiovascular events. J Nucl Cardiol.

[11] Vlachopoulos C., Terentes-Printzios D., Katsaounou P. (2022). Time-related aortic inflammatory response, as assessed with 18F-FDG PET/CT, in patients hospitalized with severely or critical COVID-19- the COVAIR study. J Nucl Cardiol.

[12] Yu A.F., Jones L.W. (2015). Modulation of cardiovascular toxicity in Hodgkin lymphoma: potential role and mechanisms of aerobic training. Future Cardiol.

[13] Vlachopoulos C., Koutagiar I., Skoumas I. (2019). Long-term administration of proprotein convertase subtilisin/kexin type 9 inhibitors reduces arterial FDG uptake. J Am Coll Cardiol Img.

[14] Mäki-Petäjä K.M., Elkhawad M., Cheriyan J. (2012). Anti-tumor necrosis factor-α therapy reduces aortic inflammation and stiffness in patients with rheumatoid arthritis. Circulation.

[15] Vlachopoulos C.V., Koutagiar I.P., Georgakopoulos A.T. (2020). Lymphoma severity and type are associated with aortic FDG uptake by 18F-FDG PET/CT imaging. J Am Coll Cardiol CardioOnc.

[16] Faeh D., Braun J., Bopp M. (2012). Body mass index vs cholesterol in cardiovascular disease risk prediction models. Arch Intern Med.

[17] D’agostino R.B., Vasan R.S., Pencina M.J. (2008). General cardiovascular risk profile for use in primary care. Circulation.

[18] Cheson B.D., Fisher R.I., Barrington S.F. (2014). Recommendations for initial evaluation, staging, and response assessment of Hodgkin and non-Hodgkin lymphoma: the Lugano classification. J Clin Oncol.

[19] Toutouzas K., Skoumas J., Koutagiar I. (2018). Vascular inflammation and metabolic activity in hematopoietic organs and liver in familial combined hyperlipidemia and heterozygous familial hypercholesterolemia. J Clin Lipidol.

[20] Brili S., Oikonomou E., Antonopoulos A.S. (2018). 18F-Fluorodeoxyglucose positron emission tomography/computed tomographic imaging detects aortic wall inflammation in patients with repaired coarctation of aorta. Circ Cardiovasc Imaging.

[21] Lawal I.O., Orunmuyi A.T., Popoola G.O. (2020). FDG PET/CT for evaluating systemic arterial inflammation induced by anthracycline-based chemotherapy of Hodgkin lymphoma: a retrospective cohort study. Medicine (Baltimore).

[22] Gholiha A.R., Hollander P., Glimelius I. (2021). Revisiting IL-6 expression in the tumor microenvironment of classical Hodgkin lymphoma. Blood Adv.

[23] van Nimwegen F.A., Schaapveld M., Janus C.P. (2015). Cardiovascular disease after hodgkin lymphoma treatment 40-year disease risk. JAMA Intern Med.

[24] Abuamsha H., Kadri A.N., Hernandez A.V. (2019). Cardiovascular mortality among patients with non-Hodgkin lymphoma: differences according to lymphoma subtype. Hematol Oncol.

[25] Rominger A., Saam T., Wolpers S. (2009). 18F-FDG PET/CT identifies patients at risk for future vascular events in an otherwise asymptomatic cohort with neoplastic disease. J Nucl Med.

[26] Figueroa A.L., Abdelbaky A., Truong Q.A. (2013). Measurement of arterial activity on routine FDG PET/CT images improves prediction of risk of future CV events. J Am Coll Cardiol Img.

[27] Marnane M., Prendeville S., McDonnell C. (2014). Plaque inflammation and unstable morphology are associated with early stroke recurrence in symptomatic carotid stenosis. Stroke.

