# Supplementary material for: Effects of Chemotherapy on Aortic 18-Fluorodeoxyglucose Uptake in Patients With Hodgkin and Non-Hodgkin Lymphoma
Source: JACC Adv. 2023 Mar 31;2(2):100277. doi: 10.1016/j.jacadv.2023.100277 (PMC11198565; doi:10.1016/j.jacadv.2023.100277)
Supplement: Supplementary Data [file mmc1.docx]

# SUPPLEMENTAL APPENDIX

# PET CT image analysis intra and inter-class correlation coefficients

Intra- and inter-class correlation coefficients (ICCs) were computed to evaluate the intra- and inter- observer reproducibility of TBR. Based on previous studies [1] we considered that a number of 15 cases would be enough for the reliability analysis. To further substantiate the lack of variability, we included 17 cases that were selected randomly . Investigator 1 and 2 analyzed the images of these cases independently. All images were re-analyzed by investigator 1 and investigator 2 two weeks later, and TBR values with ICC lower than 0.80 were considered as of poor reproducibility.

Intra-correlation coefficients (ICCs) with 95% confidence intervals were calculated to test the intraobserver variability (2-way random effects model with absolute agreement), and also to assess interobserver agreement (2-way mixed effects model with absolute agreement) for TBR assessment.

For investigator 1 the average measure intra-class correlation coefficient (ICC) was 0.997 with a 95% confidence interval from 0.991 to 0.999 p=0.0001. For investigator 2 the average measure ICC was 0.993 with a 95% confidence interval from 0.981 to 0.997, p=0.0001. The interrater reliability between investigator 1 and investigator 2 was strong with a 95% confidence interval from 0.913 to 0.989, p=0.001 (supplemental Figure 1).

**Supplemental Figure 1:** Demonstration of intraobserver and interobserver agreement for aortic TBR assessment


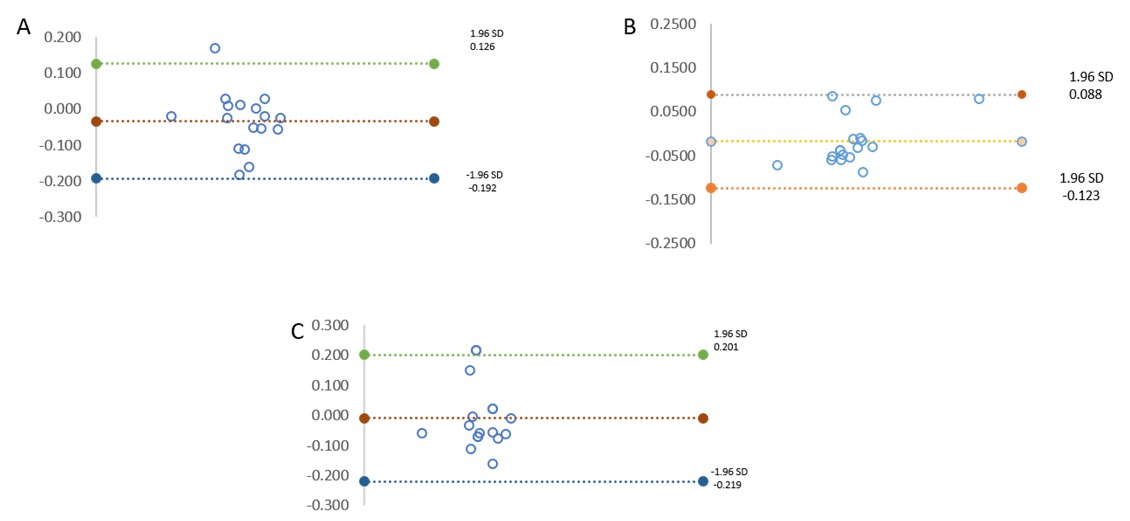


Bland Altman Plots demonstrating A: reader 1 intraobserver agreement. B: reader 2 intraobserver agreement. C: reader 1 and reader 2 interobserver agreement

**Supplemental Figure 2**: A. Global aortic TBR change during and post 1st line treatment in all patients with HL and NHL B. Global aortic TBR change during and post 1^st^ line treatment excluding patients that did not respond to first line treatment

**A**. Significant reduction of global aortic TBR in patients with Hodgkin lymphoma before and after completion of first line treatment while there is no significant response in global aortic TBR in patients with non-Hodgkin lymphoma **B**. Global aortic TBR reduction remained significant after excluding patients with incomplete metabolic response to 1st line treatment in patients with HL and remained non-significant in patients with NHL.

**GLA- TBR analysis excluding outliers**

**A. Overall population**

There was a statistically significant reduction in GLA-TBR (median GLA- TBR_baseline_: 1.92 (25th-75^th^ percentile 1.70-2.16), median GLA- TBR_3rdscan_: 1.74 (25th-75th percentile 1.59-1.93) when comparing baseline to end of treatment values, p=0.001. GLA-TBR change during treatment in patients with lymphoma is demonstrated in **Sup Figure 3.**

**B. Patients with HL**

Ιn the group of patients with HL, there was a statistically significant reduction in GLA-TBR (median GLA- TBR_baseline_: 1.88 (IQR 1.65-2.16), median GLA-TBR_3rdscan_: 1.68(IQR 1.53-1.91)) when comparing baseline to end of treatment scans, p=0.005).

**C. Patients with NHL**

In the group of patients with NHL there was a borderline non-significant reduction in GLA – TBR (median GLA-TBR_baseline_: 1.92 (IQR 1.74-2.17), median GLA-TBR_3rdscan_: 1.76(IQR 1.65-1.99)) when comparing baseline to end of treatment scans, p=0.502). The HL and NHL box plots exluding the outliers from our initial analysis is demonstrated in **Supplement Figure 4.**

**Supplement Figure 3:**

Box plot of Median GLA-TBR at baseline during and post 1st line treatment at patients with Lymphoma excluding outliers.


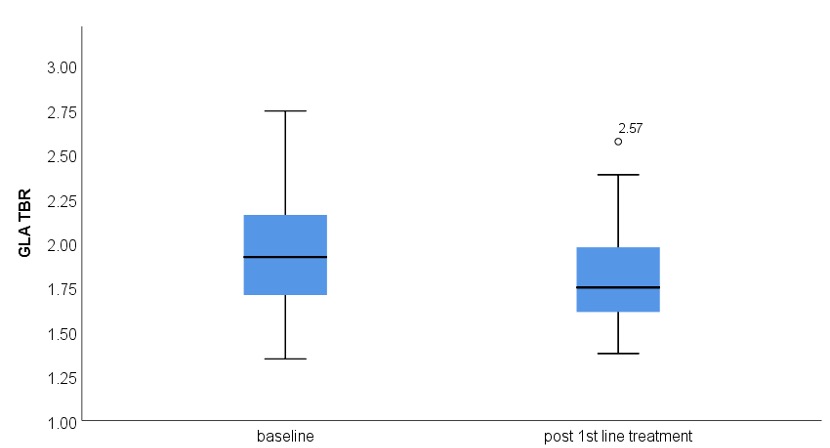


Significant reduction of global aortic TBR in patients with lymphoma before and after completion of first line treatment. TBR: target to background ratio

**Supplement Figure 4:** Box plot of GLA-TBR change between baseline and post 1st line treatment in patients with HL and NHL


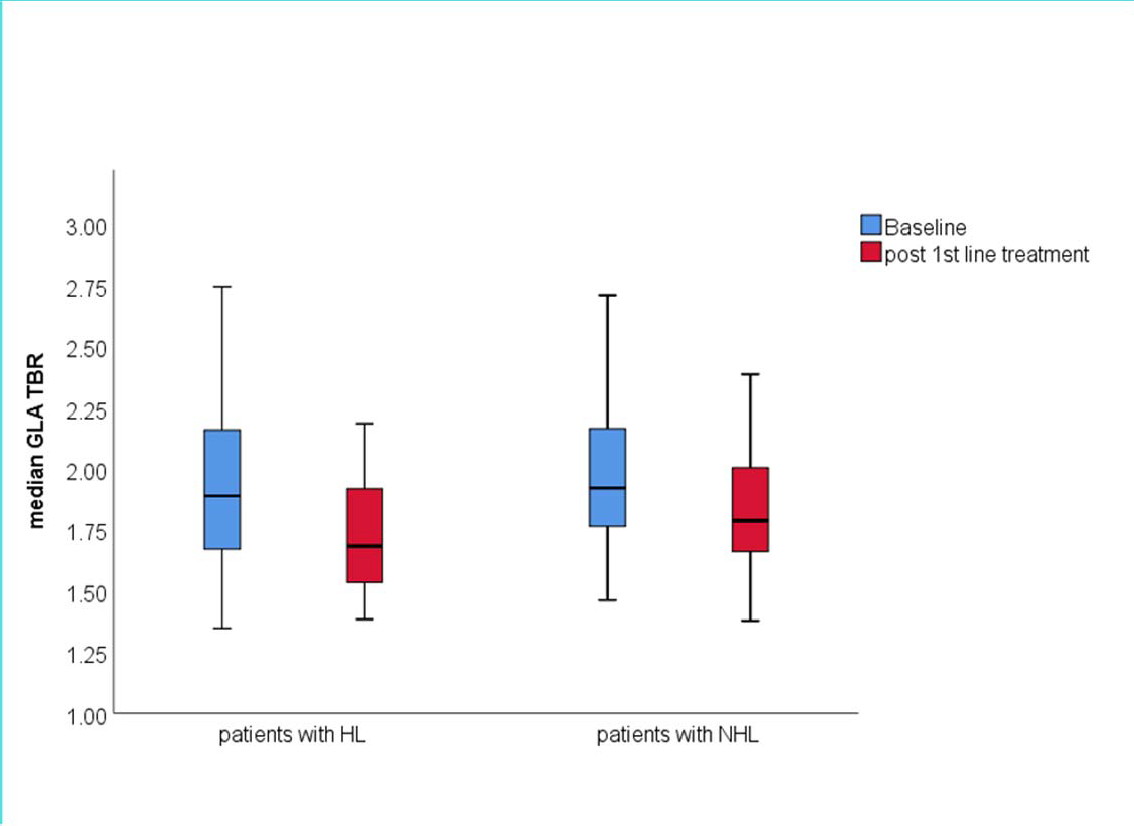


**A**. Significant reduction of global aortic TBR in patients with HL before and after completion of first line treatment while there is no significant change in patients with NHL. GLA-TBR:global aortic - target to background ratio

**References**

1. Charalambos V. Vlachopoulos, Iosif P. Koutagiar, Alexandros T. Georgakopoulos, Anastasia G. Pouli et al, Lymphoma Severity and Type Are Associated With Aortic FDG Uptake by 18F-FDG PET/CT Imaging, J Am Coll Cardiol CardioOnc. 2020 Dec, 2 (5) 758–770
